# Analysis of Oxidative Stress Enzymes and Structural and Functional Proteins on Human Aortic Tissue from Different Aortopathies

**DOI:** 10.1155/2014/760694

**Published:** 2014-07-01

**Authors:** María Elena Soto, Elizabeth Soria-Castro, Verónica Guarner Lans, Eleazar Muruato Ontiveros, Benjamín Iván Hernández Mejía, Humberto Jorge Martínez Hernandez, Rodolfo Barragán García, Valentín Herrera, Israel Pérez-Torres

**Affiliations:** ^1^Immunology Department, National Institute of Cardiology “Ignacio Chavez”, Juan Badiano 1, Sección XVI, Tlalpan, 14080 Mexico City, DF, Mexico; ^2^Pathology Department, National Institute of Cardiology “Ignacio Chavez”, Juan Badiano 1, Sección XVI, Tlalpan, 14080 Mexico City, DF, Mexico; ^3^Physiology Department, National Institute of Cardiology “Ignacio Chavez”, Juan Badiano 1, Sección XVI, Tlalpan, 14080 Mexico City, DF, Mexico; ^4^Cardiovascular Surgery Department, National Institute of Cardiology “Ignacio Chavez”, Juan Badiano 1, Sección XVI, Tlalpan, 14080 Mexico City, DF, Mexico

## Abstract

The role of oxidative stress in different aortopathies is evaluated. Thirty-two tissue samples from 18 men and 14 women were divided into: 4 control (C) subjects, 11 patients with systemic arterial hypertension (SAH), 4 with variants of Marfan's syndrome (MV), 9 with Marfan's syndrome (M), 2 with Turner's syndrome, and 2 with Takayasu's arteritis (TA). Aorta fragments were homogenized. Lipoperoxidation (LPO), copper-zinc and manganese superoxide dismutase (Mn and Cu-Zn-SOD), catalase (CAT), glutathione peroxidase (GPx), glutathione S-transferase (GST), endothelial nitric oxide synthase (eNOS), nitrates and nitrites (NO_3_^−^/NO_2_^−^), and type IV collagen, and laminin were evaluated. There was an increase in Mn- and Cu-Zn-SOD activity in SAH, MV, M, and Turner's syndrome. There was also an increase in CAT activity in M and Turner' syndrome. GPx and GST activity decreased and LPO increased in all groups. eNOS was decreased in SAH, MV, and M and NO_3_^−^/NO_2_^−^ were increased in SAH and TA. Type IV collagen was decreased in Turner's syndrome and TA. Laminin *γ*-1 was decreased in MV and increased in M. In conclusion, similarities and differences in oxidative stress in the different aortopathies studied including pathologies with aneurysms were found with alterations in SOD, CAT, GPx, GST, and eNOS activity that modify subendothelial basement membrane proteins.

## 1. Introduction

Oxidative stress is caused by an imbalance between the production of reactive oxygen species (ROS) and the antioxidant capacity of the biological system. It requires rapid detoxification of intermediate reactants or the repair of the damage and it alters the essential processes possibly becoming the origin of tissue damage in the organism [1–3].

Aortic pathologies, characterized by the loss of contractile function and endothelium-mediated relaxation, are related to oxidative stress and vascular dysfunction [4–6]. Besides, the mechanical properties of the vessel transform [7], leading to pseudoaneurysm or aneurysm formation, to obstruction or destruction of the vessel [8, 9]. Even though the origin of aortic damage might be multifactorial, molecular oxygen could play a critical role in vascular tonicity, cardiac contractility, and other parameters. Molecular oxygen participates in ROS genesis and/or can induce irreversible damage and even death. However, it can also have beneficial effects, participating in cellular signaling processes [10]. Oxidative stress has been involved in cardiovascular diseases [2, 11, 12], such as arrhythmias, coronary arterial disease, left ventricular hypertrophy, aortic dilatation, aortic dissection, and congestive heart failure. ROS and reactive nitrogen species (RNS) are produced in these diseases through different pathways such as mitochondrial xanthine oxidase and NADPH oxidase (Nox). Endothelial nitric oxide synthase (eNOS) also plays a relevant role in them [13–24].

Structural and functional damage is present in the aortic walls in different human diseases such as hypertension (SAH), atherosclerosis, Takayasu's arteritis (TA), Turner's syndrome, and Marfan's syndrome (M) and its variants (MV) [25–28]. In them, aortic damage, stenosis, occlusion, aneurysms or pseudoaneurysms [29–31], and endothelial dysfunction [32–34] have been described. These findings have been described independently for each disease having a specific genetic background, appearing at different ages and in subjects of different genders and exposed to different environments such as infections or trauma. However, they all require similar surgical treatment [35].

The role of oxidative stress in aortic damage progression in some pathologies has already been described [7, 36]; however, in others, human tissues are scarce [37, 38]. Therefore, a study of oxidative stress and antioxidants in the aorta from patients with aortic damage is justified.

The aim of this paper is to evaluate the role of oxidative stress in human aortas from patients with different pathologies where aortic damage is present. Tissue was obtained by elective surgery or emergency. We also evaluate subendothelial basement membrane proteins.

## 2. Materials and Methods

### 2.1. Patients

18 men and 14 women treated at our institution and who met a surgical criteria for either elective or emergency surgery by Bentall and Bono's method [39] were included in a consecutive way as soon as aortic root dilatation (>50 mm) was demonstrated by computed tomography angiography.

The samples collected were taken from patients with M, classified by the Ghent's criterion in 1996 [40], and TA diagnosed by the standards of the American College of Rheumatology (ACR) [41] and patients with hypertension (SAH) and Turner's syndrome [42]. Once patients fulfilled the inclusion criteria they went through a thorough clinical examination to determine the extent of their cardiac pathology. The results were analyzed and presented in a clinic and pathological conference where cardiovascular specialists assessed the risks and benefits of a surgical intervention. The subjects were submitted to a preoperative protocol that included coagulation tests, X-rays, electrocardiogram, anesthesia evaluation, and individualized medical intervention. Cases were dealt with caution, to avoid including patient undertaking treatments with antioxidants, allopurinol, or probable inhibitors of pathways involved in ROS production. Aspirin, warfarin, clopidogrel, and other antiplatelet or anticoagulant medications were suspended. Control tissues were obtained from patients who underwent surgery for aortic stenosis, who had no syndromic pathology diagnosed, and in whom there was no suspicion of inflammatory diseases such as TA or atherosclerosis nor presence of degenerative disorders such as diabetes mellitus, arterial hypertension, thyroid, or autoimmune diseases. The surgery performed implied substitution of aortic valves and there was a need to perform plasty or resection of aortic tissue surrounding the valvular area. Control subjects were evaluated previously to surgery by an expert cardiologist and rheumatologist to verify that none of the above mentioned diseases was present and routine laboratory tests were made to determine acute phase reactants, triglycerides, and HDL cholesterol. Additionally to image studies by echocardiography, computerized tomography or magnetic resonance studies were done to discard aortic damage additional to valvular damage. None of the control subjects was taking anti-inflammatory drugs or statins. The research protocol was approved by the Research and Ethics Committee of our institution (Institutional protocol number: 09654). Informed consent of patients and controls was obtained for the anesthetic procedure and surgery and to obtain a tissue sample, according to the Declaration of Helsinki [43]. Once the surgery was performed, the tissue was placed in liquid nitrogen and was kept at −70°C until use.

### 2.2. Thoracic Aorta Homogenization

A sample from thoracic aorta was taken for homogenization in liquid nitrogen, it was mixed with a sucrose solution in the presence of protease inhibitors (1 mM PMSF, 2 *μ*M pepstatin A, 2 *μ*M leupeptin, and 0.1% aprotinin), and the preparation was kept on ice. The thoracic aorta homogenate was centrifuged at 900 ×g for 10 min at 4°C. The supernatant was separated and kept at −70°C until required. Protein concentration in the thoracic aorta homogenate was determined by the method of Lowry et al. [44].

### 2.3. Immunofluorescence

3 mm of aortic sections was quickly frozen in Tissue-Tek (Sakura Finetek USA, Inc., Torrance, CA). Sections were fixed with acetone and were blocked with PBS/azide 0.02%/BSA 1% for 30 min.

Subsequently, these sections were left for 2 hours at room temperature with a rabbit polyclonal antibody against type IV collagen (1 : 20), fibronectin, laminin-*γ*-1, iNOS, and eNOS (1 : 50; Santa Cruz Biotechnology, Inc., Santa Cruz, CA). Primary antibodies were detected with goat, anti-rabbit, FITC (Jackson ImmunoResearch Laboratories Inc., West Grove, PA), at room temperature for 60 min. Negative controls were prepared by substituting the primary antibody with an irrelevant antibody. Immunofluorescence was examined using a fluorescence microscope (Nikon Eclipse TE2000-U, Digital Nikon sight chamber D5-U3). Images were processed with SigmaScan Pro program, Image Analysis version 5.0.0, 1987–1999 SPSS Inc.

### 2.4. Lipoperoxidation (LPO)

LPO, a marker of damage by free radicals, was measured by a standard method [45]. One mg of protein from the thoracic aorta homogenate was used.

### 2.5. Superoxide Dismutase (SOD) Activity

SOD enzyme activity was determined in the thoracic aorta homogenate by nondenaturing gel electrophoresis and nitroblue tetrazolium staining as described by Flohe and Otting [46]. 60 *μ*g of the thoracic aorta homogenate was used. SOD activity was 5600 units/mg, calculated following the technique described by Pérez et al. [45]. Riboflavin and TEMED in the presence of UV light and oxygen produce ROS; nitroblue tetrazolium and SOD compete with them. Where SOD is present, the gel remains transparent, whereas reduced nitroblue tetrazolium turns it into purple-blue.

### 2.6. Catalase (CAT) Activity

60 *μ*g of thoracic aorta homogenate was analyzed by native-gel electrophoresis with 8% polyacrylamide [47]. Protein CAT activity was 25000 units/mg, calculated following the technique described by Pérez et al. [45]. The gels of CAT and SOD were analyzed by densitometry by the image analyzer with SigmaScan Pro program, Image Analysis version 5.0.0, 1987–1999 SPSS Inc.

### 2.7. Assay of Glutathione S-Transferase (GST)

The specific activity of cytosolic GST was determined spectrophotometrically at 340 nm by the method of Beutler [48]. 100 *μ*g from aorta homogenate was used per sample. The specific activity of GST is expressed in micromoles of CDNB-GSH conjugate formed/min/mg protein.

### 2.8. Assay of Glutathione Peroxidase (GPx)

The enzyme GPx activity was measured spectrophotometrically at 340 nm by the method of Flohé and Günzler [49]. 100 *μ*g from aorta homogenate was used per sample.

### 2.9. Nitrates and Nitrites

Nitrates and nitrites were measured spectrophotometrically at 540 nm by the method described by Pérez et al. [45]. 100 mg of protein from aorta homogenate was used per sample.

### 2.10. Immunoblotting

100 *μ*g from aorta homogenate was used per sample. The immunoblotting was done according to the method described by Pérez et al. [45]. A dilution of 1/500 of the rabbit primary IgG polyclonal antibodies against eNOS, iNOS, laminin-*γ*-1, and COL4A2 (NOS3 antibody rabbit IgG [C-20]: sc-654, NOS2 antibody rabbit IgG [C-19]: sc-649, laminin-*γ*-1 antibody rabbit IgG [H-190]: sc-5584, and COL4A2 antibody rabbit IgG [N-14]: sc-70244; Santa Cruz Biotechnology, Inc.) was used.

### 2.11. Statistical Analysis

A descriptive analysis was performed. Univariate test includes age, gender, pathology types, and variables, and dichotomic and nominal variables were described as relative frequencies (percentages) and compared by chi-squared distribution or Fisher's exact test, as convenient. For bivariate analysis, continuous quantitative variables of normal distribution were compared by* t* student, and nonparametric ones by Mann-Whitney* U* test.

## 3. Results

### 3.1. General Characteristics

Out of the 32 patients, 14 were women, and 18 were men. The demographical characteristics of the patients are shown in Table 1, and the clinical data are shown in Table 2.

Measurements were made individually and they were grouped afterwards to be analyzed.

### 3.2. Lipoperoxidation

**Figure 1:**
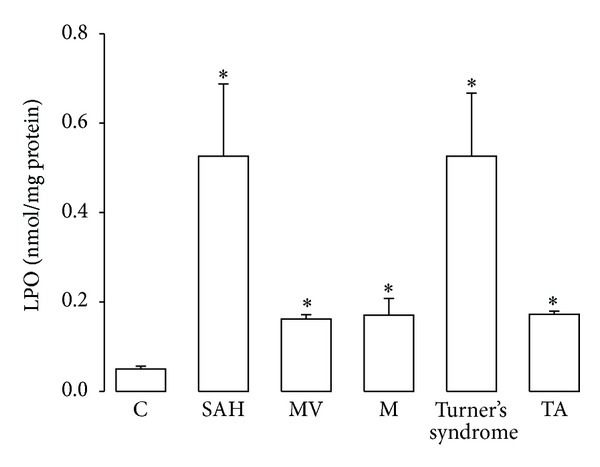
Comparison of LPO results in control subjects and in patients with the different pathologies studied. *P ≤ 0.05; C versus SAH, MV, M, Turner's syndrome, and TA. Abbreviations: SAH: systemic arterial hypertension; MV: variants of Marfan's syndrome; M: Marfan's syndrome; Turner's syndrome; and TA: Takayasu's arteritis.

Aortic tissues from patients with SAH, MV, M, Turner's syndrome, and TA showed significantly increased LPO (*P* ≤ 0.05), when compared to C subjects (Figure 1).

### 3.3. Activity of Antioxidant Enzymes

**Figure 2:**
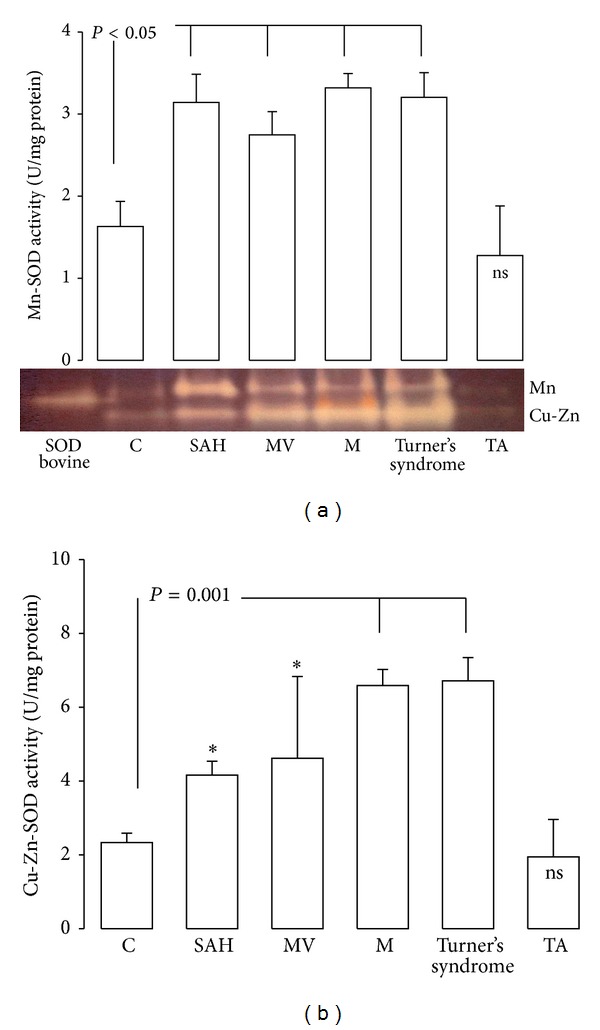
Mn-SOD and Cu-Zn-SOD activity in control subjects and in patients with the different pathologies studied. *P ≤ 0.05; C versus MV and SAH.

**Figure 3:**
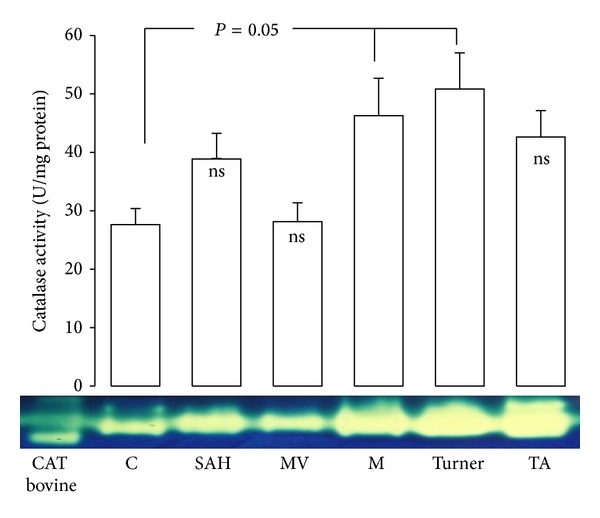
Catalase activity in control subjects and in patients with the different pathologies studied.

**Figure 4:**
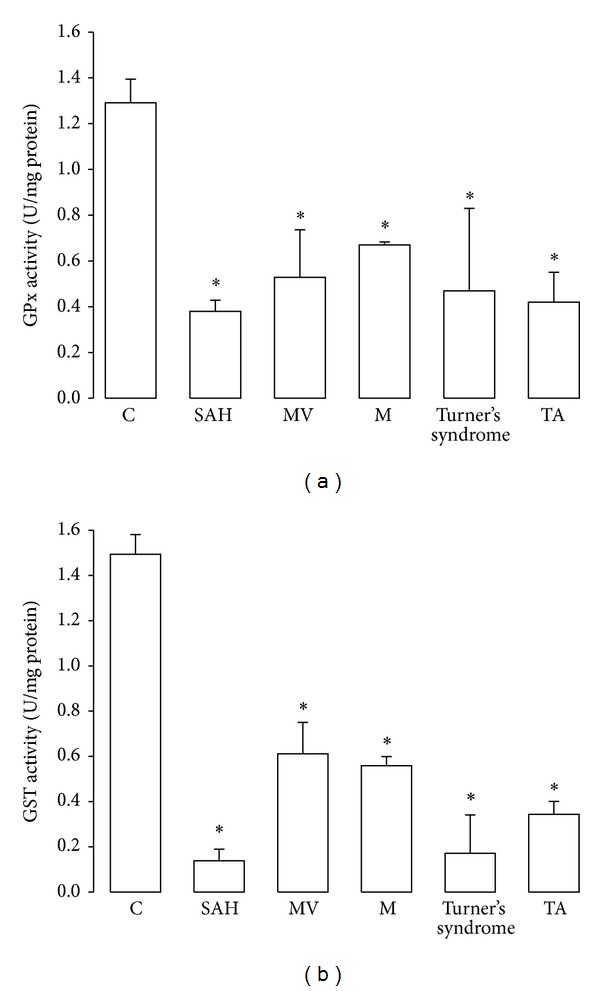
Glutathione peroxidase and glutathione S-transferase activity in controls and in the pathologic conditions studied. *P = 0.05, C versus SAH, MV, M, Turner's syndrome, and TA. Abbreviations: SAH: systemic arterial hypertension, MV: variants of Marfan's syndrome, M: Marfan's syndrome; Turner's syndrome; and TA; Takayasu's arteritis.

In SAH, MV, M, and Turner's syndrome, aortas showed an increase in Mn-SOD activity and Cu-Zn-SOD (*P* < 0.05) in comparison to C subjects (Figures 2(a) and 2(b)). CAT activity increased in M and Turner's syndrome patients (*P* = 0.05) in comparison to C subjects (Figure 3).  Figure 4(a) shows the GPx activity in SAH, MV, M, Turner's syndrome, and TA patients which was significantly increased (*P* < 0.05) when compared to C subjects.  Figure 4(b) shows the GST activity in SAH, MV, M, Turner's syndrome, and TA. It was significantly increased in these groups (*P* < 0.05) when compared to C subjects.

### 3.4. Endothelial Nitric Oxide Synthase and NO_3_^**−**^/NO_2_^**−**^

**Figure 5:**
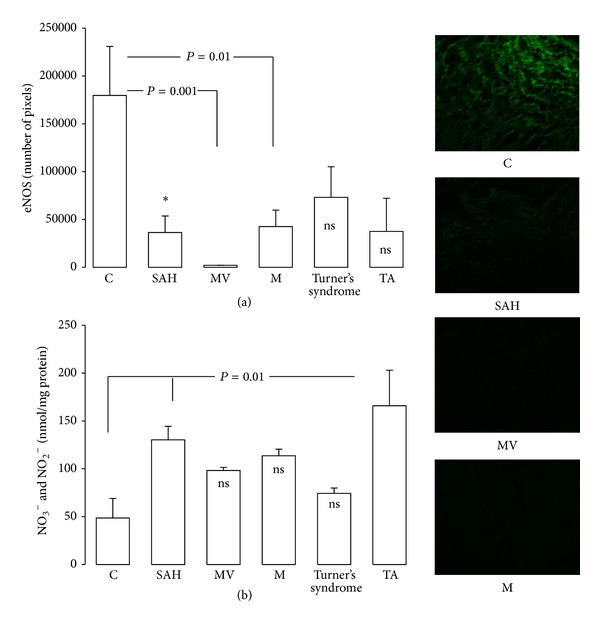
Comparison of the eNOS activity and NO_3_^−^/NO_2_^−^ in control subjects and in patients with the different pathologies studied *P = 0.05, C versus SAH. Pictures show the eNOS immunofluorescence that were significantly different.

In patients with SAH, MV, and M, eNOS expression was elevated (*P* = 0.01) in comparison to C subjects (Figure 5(a)). NO_3_^−^/NO_2_^−^ significantly increased in SAH and TA (*P* = 0.01) in comparison to C subjects (Figure 5(b)).

### 3.5. Variations in Structural and Functional Proteins

**Figure 6:**
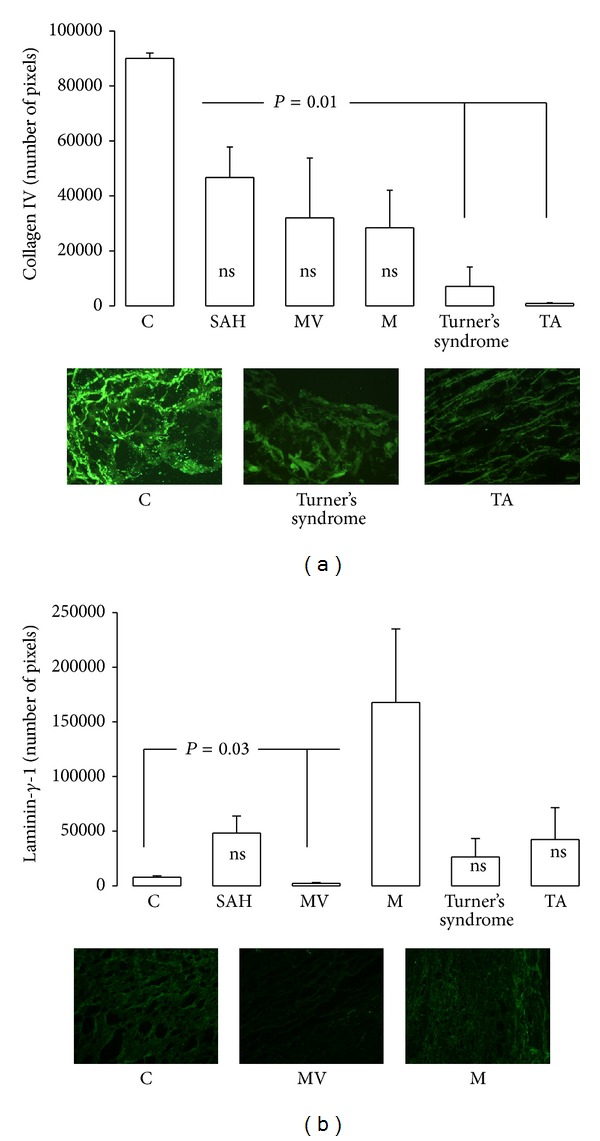
Immunofluorescence results for collagen and laminin-*γ*-1 in control subjects and in patients with the different pathologies studied. Pictures show type IV collagen and laminin-*γ*-1 immunofluorescence with statistically significant differences.

Figure 6(a) shows the localization of type IV collagen in aortas from patients. A decrease in type IV collagen was observed in Turner's syndrome and TA (*P* = 0.01) when compared to C subjects. Fibronectin did not show changes in any group (results not shown).  Figure 6(b) shows that laminin-*γ*-1 in aorta of MV was decreased while in M patients it was increased (*P* = 0.03) when compared to C subjects.

### 3.6. Inducible Nitric Oxide Synthase

**Figure 7:**
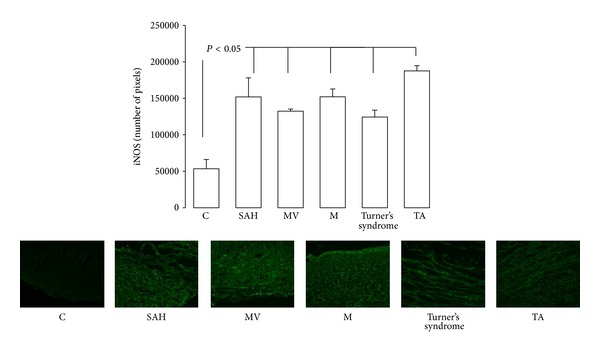
Immunofluorescence results and comparison of the iNOS expression in control subjects and in patients with the different pathologies studied. Pictures show the iNOS immunofluorescence with significant differences.

In patients with SAH, MV, M, Turner syndrome, and TA iNOS expression was elevated (*P* < 0.05) in comparison to C subjects (Figure 7).

### 3.7. Immunoblotting

**Figure 8:**
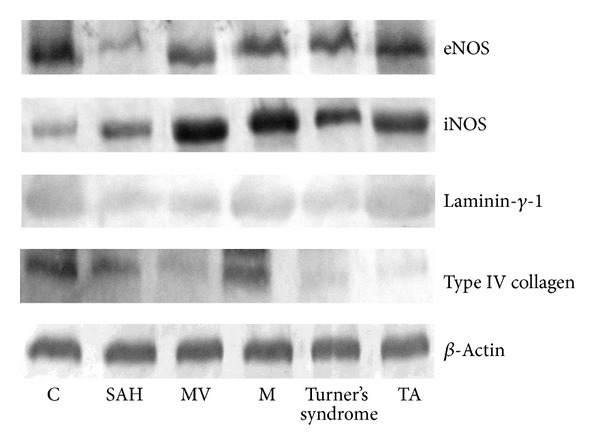
eNOS, iNOS, type IV collagen, and laminin-*γ*-1 expression in homogenized aorta. Abbreviations: SAH: systemic arterial hypertension, MV: variants of Marfan's syndrome, M: Marfan's syndrome, Turner's syndrome, and TA: Takayasu's arteritis.

Figure 8 shows eNOS, iNOS, type IV collagen, and laminin-*γ*-1 expression in the homogenized aorta from the patients with aortopathies. The eNOS, iNOS, type IV collagen, and laminin-*γ*-1 expression showed the same trend as the one found by immunofluorescence.

## 4. Discussion

The mechanisms through which oxidative stress and inflammatory processes might produce vascular abnormalities are unknown. In this paper we studied variations in the activity of antioxidant enzymes and in structural and functional proteins in several illnesses in which there is vascular dysfunction and altered mechanical properties that lead to pseudoaneurysms/aneurysms or obstruction. The disease renders it necessary for the patients to undergo surgery. The exact source of ROS and RNS in the pathophysiological pathways of these pathologies has not been clearly described.

ROS and LPO lead to DNA oxidative damage and to high levels of 8-iso- prostaglandin F_2*α*_ (8- iso-PGF_2*α*_) in patients having essential hypertension [50]. Serum of patients with TA shows a similar tendency [51]. The role of ROS in the onset and progression of aortic damage has been described in animal models of different illnesses [52] and ROS are important mediators in the signaling pathways of inflammation and atherogenesis [53, 54]. An imbalance between the prooxidative agents and antioxidants leads to changes in the redox state. Mouse models for M have vasomotor dysfunction in the thoracic aorta associated with oxidative stress, which correlates with an increase in eNOS and a diminished production of Mn- and Cu-Zn-SOD [4, 55]. Our results show an increase in Mn-SOD and Cu-Zn-SOD activity in SAH, MV, M, and Turner's syndrome. The increased expression and activity found suggest that, due to the increase in oxidative stress, the patient's antioxidant system is overexpressed in an attempt to counteract the imbalance. The vascular protection by the expression and activity of the isoforms of the SOD in the vascular wall has been evaluated in mouse models, finding either SOD deficiency or overproduction [56]. An increase in vascular permeability or reperfusion injury after ischemia is related to SOD deficiency, and the overexpression of the Cu-Zn-SOD protects against reperfusion injury [57]. Also, cytosolic and extracellular SOD expression alterations might impact on vascular and other tissue structures, because they inhibit vascular and myocardial hypertrophy [58].

The beneficial function of SOD as antioxidant in diverse illnesses [59] both in animal models and in patients with or without an active inflammatory disease has been described [60]. The mechanisms inducing inflammation may act through different stimulus and receptors among which the type 1 angiotensin receptor seems to play an important role [61]. Our results showed an increase in Mn- and Cu-Zn-SOD enzyme activity in SAH, MV, M, and Turner's syndrome without significant changes in TA when compared to C subjects. These results suggest that SOD isoforms increase their activity to protect against oxidative stress.

SOD isoforms are important within the vascular wall in normal conditions and in diseased states in humans. Cu-Zn-SOD expression is relatively high in all cell types, including blood cells, and it accounts for 50% to 80% of the total activity, being, therefore, the predominant isoform. Mn-SOD is responsible for 2% of the activity and the remaining 12% of the activity may be due to extracellular SOD (SOD-EC). The functional importance of Cu-Zn-SOD is further evidenced when there is deficiency of SOD-EC [62]. In mice with Cu-Zn-SOD deficiency there is an increase in vascular permeability and ischemia related to hypertrophy of cerebral arterioles [63]. In our study, the Cu-Zn-SOD deficiency found in hypertensive patients with aortic dilatation, Turner's syndrome, and TA could be related to vascular alterations and to the clinical condition of these patients. In genetically modified mice with Cu-Zn-SOD overexpression there is a protective effect against vascular dysfunction [64].

Under normal conditions, Mn-SOD is the first line of defense against oxidative stress. Its localization, induction mechanisms, vascular expression, and activity are known [65] and might be altered under physiological and pathophysiological conditions, particularly under overregulation of oxidative stress [66]. Mn-SOD expression is altered at certain stages of disease that are associated with vascular oxidative stress.

Proinflammatory cytokines and LPS-mediated inflammation in vascular tissue cause an increase in superoxide (O_2_^−^) production and in Mn-SOD expression [67]. In atherosclerosis, vascular expression of Cu-Zn-SOD and Mn-SOD mRNA increases at the onset of the disease and diminishes over time [68]. In chronic hypertensive models, the expression of vascular Mn-SOD is also increased [69]. There are many conditions that elevate peroxynitrite concentrations which, in turn, inactivate Mn-SOD. These include inflammation, diabetes, hypertensive atherosclerosis, subarachnoid hemorrhage, and age. However, in recent studies, Mn-SOD was found to protect from mitochondrial vascular damage and atherosclerosis development [70].

In this study, all of the aortopathy groups had an increase in Mn- and Cu-Zn-SOD, which correlated with increased LPO with the exception of TA. Mn-SOD overexpression reduces superoxide levels [71] and improves endothelial function in some models, thereby preventing endothelial injury [72]. Moreover, we consider that, in these patients, the compensatory mechanisms were diminished and oxidative stress was increased. In other vascular illnesses, these compensatory response mechanisms vary and could be associated with other factors such as evolution of the pathology, etiopathogenic mechanisms, and host response capacity [73]. TA is associated with tuberculosis infection which has only been proven in isolated cases [74–77]. In patients with confirmed tuberculosis, an important decrease in Mn-SOD has been found, which improves with treatment [78]. Recent studies proposed Mn-SOD as an important antioxidant modulator after vascular injury. These results suggest that it might be employed as a promising therapeutic strategy for vascular injury prevention and in proliferative diseases where there is stenosis [79].

CAT is another important antioxidant enzyme, which is found in the liver, kidney, and aorta. CAT uses two H_2_O_2_ molecules to break them into O_2_^−^; one acts as a reducing agent and the other as an oxidant. Overexpression of CAT prevents the stimulation of ROS [80]. Our results showed that CAT increased in the patients with M and Turner's syndrome. These results suggest that overexpression of CAT may be due to overproduction of H_2_O_2_ in the aorta from these patients. The increase in CAT activity in our study may be attributed, in part, to continuous exposure to hydroperoxides. Although different studies have shown that in hypertension CAT activity is low [81], in our study CAT activity showed no significant change in SAH, MV, and TA when compared to C subjects.

The enzyme GST conjugates GSH to electrophilic xenobiotics, chemicals, and toxic compounds like malondialdehyde which is an end product of the LPO process in phospholipids, leading to an increase in the rigidity of the cellular membrane, forming a thioether bond [82]. Patients with hypertension usually show a decrease in GST activity [83]. Our results show a decrease in GST activity in SAH, MV, M, Turner's syndrome, and TA in comparison to C subjects. A similar tendency was found in GPx activity. GPx detoxifies low levels of hydrogen peroxide with the help of GSH, causing its oxidation [84]. Hypertension is associated with decreased activity of many antioxidant enzymes including GST and GPx.

GPx can also be inactivated in conditions of oxidative stress; O_2_^−^ can inhibit the function of this enzyme [83]. These results suggest that the decrease in GST and GPx activity can be due to oxidative stress in these patients. In this study we did not analyze glutathione concentration, but the decrease in GPx and GST activity may be due in part to a decrease of glutathione and to the accumulation of ROS. When hypertension advances into stages II and III, even the defense of GPx might deteriorate because of the increased production of ROS [85].

LPO is increased in animal models, in serum from patients with atherosclerosis [86], and in TA. Our results show that LPO is increased in SAH, MV, M, Turner's syndrome, and TA, which is consistent with previous reports in the literature [87]. In essential hypertension and M an increase in the LPO levels in comparison to healthy subjects has been described [88, 89]. 8-iso-PGF_2*α*_, a marker of stress oxidative, also increases in TA [37]. Our results on MDA levels suggest that the pathologies studied show a different grade of oxidative stress with respect to C subject, modifying the activity of the antioxidant enzyme system.

The endothelium constitutively releases a number of vasoactive mediators including NO that regulate smooth muscle contractility and thus vascular smooth muscle tone and mechanical properties. Endothelial dysfunction and downregulated NO would contribute to the stiffness, the reduced distensibility, and the aortic complications that have been described in MV and M in mice models. Alteration of fibrillin-1 impairs the integrity of elastic fibers within the endothelial layer and endothelial permeability is impaired in M [90]. The reduction of NO production also decreases cGMP levels which act as downstream second messengers of NO signaling. In addition, MV and M are associated with elevated plasma levels of homocysteine, which attenuates endothelial function and limits NO bioavailability, production, and reduction. NO alterations increase the susceptibility to aortic complications [91]. Our results showed that SAH, MV, and M showed a decrease in eNOS expression. These results suggest that eNOS metabolism is decreased and that its participation is reduced in these pathologies. An explanation for the decline of eNOS is that the highly oxidative environment decreases its activity. However, our results show an increase of the NO_3_^−^ and NO_2_^−^ ratio, which are metabolites of NOS. The increase in NO_3_^−^ and NO_2_^−^ ratio may be due to inducible nitric oxide synthase (iNOS); this enzyme could be a mediator in some stages of the disease [92]. The immunofluorescence shows an increase of iNOS expression in all of the pathologies studied in comparison to control subjects. These results suggest that iNOS produces NO mainly during inflammatory processes and iNOS contributes significantly to the tissue NO_3_^−^/NO_2_^−^ ratio. It may participate in protein matrix degradation and play a causal role in aneurysm formation [93]. iNOS is widely expressed in diverse cell types that are under transcriptional regulation by inflammatory mediators and has been implicated in the pathogenesis of many disorders including atherosclerosis, stroke, arthritis, and aneurisms [94].

In the pathologies studied, the presence of aneurisms contributes to endothelial dysfunction and the increase in the NO_3_^−^ and NO_2_^−^ ratio can favor the increase of peroxynitrites. In addition, altered NO bioavailability contributes to the modified vasomotion in hypertension [95] where an increased production of ROS is associated with an elevated production of peroxynitrite in coronary blood vessels. Endothelial dysfunction also promotes an increase in the generation of O_2_^−^ leading to an enhanced NO inactivation against peroxynitrites [93]. Additionally, peroxynitrites impair NO production through oxidation of BH_4_, a NOS cofactor [96]. Furthermore elevated peroxynitrites are associated with elevations in the myogenic tone, vasoconstriction, and deterioration of the endothelium-mediated relaxation [64].

Our results show significant changes in some proteins of the extracellular matrix such as laminin-*γ*-1 and type IV collagen. A decrease in these proteins could explain, at least in part, the deterioration of vascular mechanical function in these patients. Oxidative stress could deteriorate the endothelium and favor the synthesis of subendothelial proteins. In animal models, changes in the signaling pathways promote alterations such as proliferation, migration, and remodeling of the extracellular matrix in vascular smooth muscle cells having as a consequence an increase in vascular wall thickness, inflammation, and susceptibility to develop atherosclerosis. Some animal models have been proposed to explain the structural changes in vascular pathology [52]. However, it is not clear if these mechanisms operate in a similar way in human tissues [97].

Finally, this study has, as an important limitation, the use of aortas from patients with rare conditions. This renders impractical the monitoring of each patient prospectively for a long time. The retrospective study only allows the evaluation of some aspects but does not give the opportunity to correct some biases. Prospective studies should be undertaken in a systematic way to evaluate several aspects such as the role of the oxidative stress, antioxidant therapy, and participation of factors in reducing aortic dilatation.

The relevance of this study lies in the presence of aneurysms and cardiovascular damage as an outcome in all of the conditions studied. It stimulates research interest in intervention maneuvers and preventive aspects. The study generates hypothesis from the genetic, environmental, and therapeutical points of view. The major limitation of this study is the small size of the aortic sample in diseases like MV, M, Turner's syndrome, and TA that occur with an incidence of 2-3 per 10000 individuals, being autosomal dominant disorders of the connective tissue caused by mutations. Furthermore, the causal associations between cellular and mechanical processes in the formation of aortic aneurysms have not been completely defined, so that a specific therapy has not been proposed. However, some studies have shown that simvastatin decreases free radicals, NF-*κ*B, and improves the antioxidant condition [98]. In this study, patients with M had an increase in CAT that could be associated with laminin, but this could be due to the fact that they were receiving simvastatin. In some cases with TA, hypertension, and myocardial ischemic injury, LPO was increased and antioxidant enzymes decreased, suggesting an increase in the production of ROS. In this group none of the patients received simvastatin; this observation sets up a possible future therapeutic hypothesis in these cohorts.

## 5. Conclusions

These preliminary findings show similarities and differences in the role of oxidative stress in the pathologies studied. It is necessary to implement appropriate studies and methodological strategies to assess oxidative stress in each condition, as each pathogenesis can influence the cellular redox state. Prooxidant damage mechanisms seem to be specific and a common pathway for injury and aortic deterioration. Compensatory mechanisms, in chronic stages of aortic damage, are inversely related, since in the presence of LPO there is a low antioxidant activity. However, in early stages, prooxidant and antioxidant agents seem to develop in parallel, as a response to the imbalance. In these pathologies, whose ultimate damage is the aorta, therapeutic maneuvers acting upon antioxidants should be started since diagnosis, independent of the cause of aortic damage. The cohort design, retrospective and prospective, should be appropriate for each group.

## Figures and Tables

**Table 1:** Demographic characteristics of the patients.

Characteristics	Control *n* = 4	SAH *n* = 11	Marfan's variants *n* = 4	Classic Marfan *n* = 9	Turner's syndrome *n* = 2	Takayasu's arteritis *n* = 2
Median age (range)	59 (42−62)	59 (34−72)	46 (28−55)	30 (17−56)	33 (31−34)	33 (26−40)
Women *n* (%)	1 (25)	4 (37)	1 (25)	4 (44)	2 (100)	2 (100)
Men *n* (%)	3 (75)	7 (64)	3 (75)	5 (56)	0	0
Median BMI (range)	22 (18−24)	31 (30−32)	24 (23−32)	22 (17−30)	28 (23−32)	19 (16−23)
Median LVEF (range)	44 (30−65)	45 (25−65)	60 (56−75)	50 (40−65)	59 (50−67)	65 (65-65)
Median aortic diameter (range)	55 (55-55)	52 (26−65)	66 (62−70)	75 (55−120)	81 (67−96)	50 (50-50)
SAH *n* (%)	1 (50)	11 (100)	1 (25)	2 (22)	2 (100)	1 (50)
Tobacco smoking *n* (%)	1 (50)	4 (37)	2 (100)	2 (22)	0	1 (50)

SAH: systemic arterial hypertension, BMI: body mass index, and LVEF: left ventricular ejection fraction.

**Table 2:** Clinical finding, surgery type, and report diameters aortic.

Sex	Age	Diagnosis	Clinical findings	Aortic diameter mm
H	68	SAH	SVAo and aortic reduction plastic	26
M	55	SAH	Aortic dissection and Ao. I (BB)	55
H	63	SAH	Hypertensive cardimyopathy and abdominal aneurysm (BB)	55
H	59	SAH	Aortic arc substitution and stent subclavian artery (BB)	69
M	53	SAH	Aortic root aneurysm (BB)	50
H	46	SAH	B and B aortic arc substitution and revascularization of brachiocephalic trunk	55
M	59	SAH	Ascending aorta dissection (BB)	60
M	55	SAH	Aortic valve substitution + plastia aorta + Revascularization Coronary: Internal thoracic artery-DA, Venous Hemoduct	65
H	34	SAH	Abdominal aneurysm (BB)	100
H	72	SAH	B and B aortic arc substitution and revascularization of brachiocephalic trunk	70
H	63	SAH	Ascending aortic aneurysm (David)	51
H	31	Marfan*ʼ*s syndrome	Ascending Ao. A, coarctation. Bivalve aorta (BB)	75
H	17	Marfan*ʼ*s syndrome	Aortic dissection and aortic insufficiency (BB)	87
H	56	Marfan*ʼ*s syndrome	Aortic dissection and aortic insufficiency (BB)	94
H	17	Marfan*ʼ*s syndrome	Aortic dissection and Ao. A (BB)	68
H	42	Marfan*ʼ*s syndrome	Aortic dissection and Ao. A (BB)	120
M	38	Marfan*ʼ*s syndrome	Aortic dissection and Ao. A (BB)	55
M	21	Marfan*ʼ*s syndrome	Aortic ascending aneurysm (BB)	67
H	23	Marfan*ʼ*s syndrome	Bicuspid aortic valve and ascending aorta A (BB)	96
H	23	Marfan*ʼ*s syndrome	Aortic root dilation and aneurysm (BB)	50
M	28	Marfan*ʼ*s variant	Ascending aorta aneurysm (BB)	88
H	46	Marfan*ʼ*s variant	Ascending aorta aneurysm (BB)	70
H	46	Marfan*ʼ*s variant	Thoracic aneurysm and ascending aorta (BB)	55
H	55	Marfan*ʼ*s variant	Ascending aortic and aortic root aneurysm (BB)	57
M	31	Turner's syndrome	Ascending aorta aneurysm and infradiaphragmatic aorta (By)	67
M	34	Turner's syndrome	Acute aortic syndrome and dissection (BB)	100
M	26	Takayasu's arteritis	Acute aortic syndrome and dissection of aneurysm (BB) SVAo	54
M	40	Takayasu's arteritis	Autopsia complications of IRCT	52
H	62	Control	Aortic stenosis	50
M	60	Control	Aortic stenosis	54
H	42	Control	Aortic stenosis	52
H	60	Control	Aortic stenosis	60

F: female, M: man, Ao. A: aortic aneurysm, SAH: systemic arterial hypertension, Ao. I: aortic insufficiency, BB: surgery Bental and Bono, David: David surgical procedure, Descending artery: DA, SVAo: aortic valve substitution.

## Abbreviations

CAT: Catalase
SOD: Superoxide dismutase
GPx: Glutathione peroxidase
GST: Glutathione S-transferase
ROS: Reactive oxygen species
eNOS: Endothelial nitric oxide synthese
iNOS: Inducible nitric oxide synthese
LPO: Lipoperoxidation.
